# Effectiveness and context dependency of social norm interventions: five field experiments on nudging pro-environmental and pro-social behavior

**DOI:** 10.3389/fpsyg.2024.1392296

**Published:** 2024-06-26

**Authors:** Daria Mundt, Marlene C. L. Batzke, Thanee M. Bläsing, Sandro Gomera Deaño, Anna Helfers

**Affiliations:** ^1^Institute for Psychology, Faculty of Human Sciences, University of Kassel, Kassel, Germany; ^2^Center for Environmental Systems Research, University of Kassel, Kassel, Germany

**Keywords:** behavioral change, social norms, field experiment, waste reduction, injunctive norm, descriptive norm, proscriptive norm, prescriptive norm

## Abstract

Social norm interventions hold the potential to change people’s behavior. Five field experiments (*N* = 1,163) examined the effects of a simple and easily realizable social norm nudge based on the social media format “Be like Bill.” The nudge consisted of a stick figure named Toni that communicated descriptive and injunctive norms regarding pro-environmental or pro-social behaviors. Nudge conditions were compared to no-intervention control conditions. Experiment 1 (*N* = 179) focused on paper towel consumption in a women’s restroom at a German university. The nudge condition used less paper towels than the control condition, *d* = 0.48. Experiment 2 (*N* = 183) replicated this result (*d* = 0.32) in a more diverse setting of a women’s restroom at a German Christmas market. Experiment 3 (*N* = 250) examined differences in the effects of prescriptive (i.e., ‘do-norm’) versus proscriptive (i.e., ‘do not-norm’) social norms on paper towel consumption again in a university women’s restroom. The effectiveness of both social norm nudge conditions was shown in comparison to the control condition (*d* = 0.46; *d* = 0.40), while the prescriptive and proscriptive social norm manipulations did not differ. Experiment 4 (*N* = 206) applied the nudging approach to the use of plastic lids in a coffee shop, where no effect was found. Finally, Experiment 5 (*N* = 345) focused on the pro-social behavior of mask wearing in a bakery toward the end of the Covid-19 pandemic restrictions in Germany. In the nudge condition, more visitors put on face masks compared to the control group, *d* = 0.39. Limitations and contextual factors regarding the applicability of our social norm nudge are discussed.

## 1 Introduction

Climate change, resource depletion, and many other environmental challenges facing humanity in the 21st century call for behavioral change. Naturally, change has to occur on many levels. Yet, large-scale global change cannot be achieved without change on the individual level, meaning individuals acting more pro-environmentally (van Valkengoed et al., 2022). Environmental psychology provides a variety of different approaches to achieving individual behavior change (Abrahamse et al., 2005; Varotto and Spagnolli, 2017). Effective, low-cost, and easy to implement are social influence approaches (Abrahamse and Steg, 2013). In particular, social norms have received a great deal of attention in environmental psychology, being effective in motivating people to change their behavior, even when people think they were not affected (Nolan et al., 2008). Presenting people social norm messages can also be considered a nudge: a small change in the decision-making environment without limiting people’s free choice (Thaler and Sunstein, 2008). Social norm nudges have been shown to be a reasonable way to facilitate individual behavior change toward pro-environmentalism and to harvest the so-called low-hanging fruits in behavior change (Bao and Lim, 2022).

The present study investigates the effectiveness of an easy-to-employ social norm nudge designed to promote pro-environmental and pro-social behavior in various real-world contexts. The nudge was tested in different versions as well as in different contexts. In doing so, we aimed for developing a social norm nudge that is ready-for-implementation. So far, situational and contextual variables have gained little attention in the nudging literature (e.g., Czajkowski et al., 2019; Grilli and Curtis, 2021; Mertens et al., 2022; Dannenberg and Weingärtner, 2023). In the present work, one specific nudge was developed and tested in different situational contexts. This allows considering the situational and contextual variables of its effectiveness. The desired outcome of the conducted studies was therefore not only a ready-to-use nudge, but also to provide practitioners with information about when and where it is effective and when not. In doing so, our study also provides valuable insights into empowering citizens to change their practices through practical and scalable behavioral science interventions.

### 1.1 Social norms and pro-environmental behavior

The power of social norms on behavioral decisions has been shown by a vast body of research and on a wide variety of behaviors, including pro-environmental behaviors such as recycling (e.g., White et al., 2009), energy conservation (e.g., Schultz et al., 2008), and littering (e.g., Keizer et al., 2008), to only name a few (for a review see Dannenberg et al., 2024). Social norms are informal behavioral rules governing everyday life, indicating what other people do or believe is “the right thing” to do. They are most commonly differentiated into descriptive and injunctive norms (Cialdini et al., 1990). A descriptive norm describes what is perceived as “normal” in an empirical sense, meaning the behaviors that other people show in a specific situation (Cialdini et al., 1990, p. 1015). An injunctive norm communicates what is considered as appropriate or inappropriate in a normative sense by other people in a specific situation. They communicate what it is that “ought” to be done (Cialdini et al., 1990, p. 1015). There is research on both types of social norms as well as research showing their individual effects on pro-environmental behavior (Smith et al., 2012). Following this, several studies showed that the combination of both descriptive and injunctive norms had the strongest behavioral effects (Nolan et al., 2008). More specifically, Lee et al. (2007) showed that the combined effect of descriptive and injunctive norms is not only additive but both norms positively interact with each other, leading to an additional benefit of combining both types of social norms. Combining both types of norms has been shown to have another positive effect. The individual presentation of descriptive norms can lead to so-called boomerang effects, meaning that individuals outperforming the communicated descriptive norm may lower their efforts to match the norm (Schultz et al., 2007, 2008). The boomerang effect disappears when combining the descriptive with an injunctive norm message. Due to the presented advantages of combining descriptive and injunctive norms, that is what we did in the present study.

Another differentiation regarding social norms is the one between prescriptive and proscriptive norms (e.g., Bergquist and Nilsson, 2016; Pavey et al., 2018). A prescriptive social norm describes what is (to be) done and is therefore also referred to as the “do-norm” (cf. Janoff-Bulman et al., 2009; Bergquist and Nilsson, 2019). A proscriptive norm refers to what is *not* (to be) done and is therefore also called a “do not-norm.” Compared to descriptive and injunctive norms, Much less is known about their different effects. While it has been shown that proscriptive norm messages lead to higher reactance levels in recipients (Pavey et al., 2021) and suggested that prescriptive norms are more abstract and therefore less effective (Janoff-Bulman et al., 2009), the studies that have tested their independent effects provide an inconclusive picture. Some showed that prescriptive norms are more effective (Bergquist and Nilsson, 2016, study 2; Pavey et al., 2021, study 2; Pavey et al., 2023, study 1), while a slight majority show that proscriptive norms are more effective (Bergquist and Nilsson, 2019, study 3; Cialdini et al., 2006; Mollen et al., 2021, study 2; Pavey et al., 2018, 2021, study 4). As the differentiation between prescriptive and proscriptive norms poses a relevant question for the practical application of norm interventions, namely how to formulate an effective norm message, it was addressed in the present work.

### 1.2 Nudging as intervention method

A simple intervention method to confront consumers with specific social norms is nudging. Nudging is an overarching term for simple interventions that aim at encouraging desirable behaviors by making small changes in the decision-making environment (e.g., Thaler and Sunstein, 2008; Moseley and Stoker, 2013; Lehner et al., 2016; Schubert, 2017). Among the various nudging techniques, social norm nudges are widely recognized as particularly powerful strategies (Lee et al., 2007; Sunstein, 2014; Byerly et al., 2018). In the pro-environmental setting, nudging interventions were employed for promoting waste reduction and recycling, energy and water conservation, sustainable consumption, and travel (Wee et al., 2021).

Nudging as an intervention method to guide consumers to achieve common goods has gained popularity for several reasons. Firstly, it guides individuals toward desired choices without restricting their freedom (Thaler and Sunstein, 2008). Thus, in contrast to legal restrictions or bans, individuals are still free in choosing between different behavioral options. Secondly, nudging is often cost-effective and easy to implement compared to more traditional policy interventions (Benartzi et al., 2017).

While numerous studies substantiate and underscore the success of nudging, there is also literature casting doubt on its overall efficacy (Maier et al., 2022), along with discussions on the circumstances under which nudging proves effectiveness (e.g., Costa-Font et al., 2014; Lehner et al., 2016; Szaszi et al., 2018). A central finding from this critical discourse concerns the limited generalizability of the individual nudge intervention, since the success of a nudge intervention depends on situational and contextual factors (e.g., Czajkowski et al., 2019; Grilli and Curtis, 2021; Mertens et al., 2022; Dannenberg and Weingärtner, 2023).

Acknowledging the limited generalizability of individual nudging interventions (e.g., Costa-Font et al., 2014; Lehner et al., 2016), our study aims at identifying the boundary conditions of the applicability of our specific social norm nudge to refine the conditions necessary for successful implementation. Since nudging remains a popular choice for policymakers and practitioners (Benartzi et al., 2017), empirical evidence is needed that guides practitioners in tailoring nudges to specific contexts and optimizing their effectiveness. With five field experiments, we offer practical insights for the design and implementation of a scalable social norm intervention.

### 1.3 The present study

With five field experiments, we investigated the effect of a humorous social norm nudge based on the social media meme “Be like Bill” (Croitoru and Nath, 2016) in three different areas of application (i.e., paper towels usage, lid use of disposable coffee cups and mask wearing). The nudge included an injunctive social norm message with a descriptive social norm.

The goal of the present study was to investigate a simple and ready-to-use intervention that can be easily integrated into existing systems to promote positive behaviors for the environment and society. Particularly in contexts where certain behaviors cannot be prohibited or avoided altogether, cost-effective interventions such as our social norm nudge are useful tools to at least reduce the negative consequences of specific behaviors. Through the implementation of five field experiments, our objective was to comprehensively investigate the specific contexts in which this behavioral nudge is effective. Field experiments are particularly important in this application field, as the results of laboratory experiments cannot always be seamlessly transferred to real-life decision-making scenarios or their effects may be comparatively weakened. Consequently, the knowledge gained from field experiments proves to be helpful for the formulation of practical recommendations (cf. DellaVigna and Linos, 2022). Further, by examining samples that differ in their demographic characteristics, we gain knowledge about potential target groups of this intervention method, since people respond differently to social norms (e.g., Czajkowski et al., 2019; Dannenberg and Weingärtner, 2023).

The first part of this study focused on reducing paper towel usage in public restrooms. With the significance of hand hygiene during and after the Covid-19 pandemic, the prospect of completely eliminating paper towels for environmental reasons appears unlikely, prompting our interest in finding simple ways to reduce individual paper towel consumption. Experiment 1 tested our social norm nudge in a female’s restroom of a university’s learning center. Experiment 2 replicated the same study design in a more heterogeneous setting, a restroom at a Christmas market. Experiment 3 investigated possible differences of the nudge when changing the social norms, i.e., proscriptive versus prescriptive norms. Experiments 4 and 5 extended our nudging paradigm to different application areas, i.e., lids of disposable coffee cups at a coffee shop with the aim of plastic waste reduction (Experiment 4) and mask wearing during the Covid-19-Pandemic as an example for pro-social behavior (Experiment 5). Table 1 summarizes the main characteristics and results of the five experiments.

**Table 1:** Summary of experiments 1–5.

Exp.	Setting	*N*	Type of sample	Dependent variable	Independent variable	Statistical significance	Effect size
1	Restroom (university)	179	female students	used paper towels	social norm vs. control	*p* < 0.001	*d* = 0.48
2	Restroom (christmas market)	183	female adults	used paper towels	social norm vs. control	*p* = 0.015	*d* = 0.32
3	Restroom (university)	250	female students	used paper towels	prescriptive norm vs. control	*p* = 0.002	*d* = 0.46
					proscriptive norm vs. control	*p* = 0.005	*d* = 0.40
					prescriptive vs. proscriptive	*p* = 0.777	*d* = 0.04
4	Coffee shop	206	adults (male and female)	used plastic lids	social norm vs. control	*p* = 0.881	*d* = 0.02
5	Bakery	345	adults (male and female)	mask wearing	social norm vs. control	*p* = 0.006	*d* = 0.39

## 2 Experiment 1

Experiment 1 examined our social norm nudge in a female’s restroom of a university’s learning center. The focus on paper towel use in public restrooms as application area for our intervention stemmed from the fact that despite being acknowledged as environmentally unfriendly (Umweltbundesamt, 1993), paper towels are widely regarded as the most hygienic option to dry hands (Hanna et al., 1996; Huang et al., 2012; Best et al., 2014). Considering the heightened importance of hand hygiene during and after the Covid-19 pandemic, eliminating paper towels in favor of the environment seems unlikely. Consequently, our interest lay in finding a simple yet effective way to reduce individuals’ paper towel consumption.

Effective nudge interventions were already found in the context of hand hygiene. For example, hand washing frequencies could be increased by showing arrows leading to sinks (Blackwell et al., 2017). A normative sign was useful to increase the use of sanitizer (Aarestrup et al., 2016). Goldstein et al. (2008) discovered that incorporating descriptive norm messages, informing guests about the prevalence of towel reuse, resulted in a higher rate of towel re-usage in hotel rooms. Concerning paper towel use, Lee et al. (2020) failed to produce a nudging-effect with their descriptive social norm nudge, including a detailed description of how to dry one’ hands with one paper towel only. Reactance of the participants was suggested as a possible explanation (cf. reactance theory by Brehm, 1966). With our humorous social norm nudge, we tried to counteract any emerging reactance through the intervention. Since previous research found the combination of descriptive and injunctive social norms to be successful in nudging people’s behavior (Schultz et al., 2007), we expected participants who were nudged by our social norm nudge to use less paper towels than participants in a control group without any nudge intervention.

### 2.1 Method

The study was preregistered before data collection at: https://osf.io/s2bqz.

#### 2.1.1 Design and sample

Our field experiment followed a one-factorial between-subjects design with the independent variable nudge (control condition vs. nudge condition) and the dependent variable amount of used paper towels.

Participants were *N* = 179 female-presenting adults (estimated age range^1^: 18 to 32 years, *M* = 23.08, *SD *= 2.39), with 89 participants in the nudge condition and 90 participants in the control condition. Our data collection included all individuals who washed their hands in the restroom, while excluding those who made multiple restroom visits during the observation period (based on experimenters’ recognition), participants who witnessed the nudge installation, individuals who used paper towels for purposes other than hand drying, and cases where the count of taken paper towels was ambiguous. Participants did not know that they were part of an experiment.

An *a priori* sample size estimation with G*Power (version 3.1.9.6, Faul et al., 2007) suggested an optimal sample size of 176 participants for a one-tailed independent *t*-test. We assumed a Type-1 error probability of 0.05, a power of 0.95 and a medium-sized effect of *d* = 0.50.

#### 2.1.2 Material

**Figure 1:**
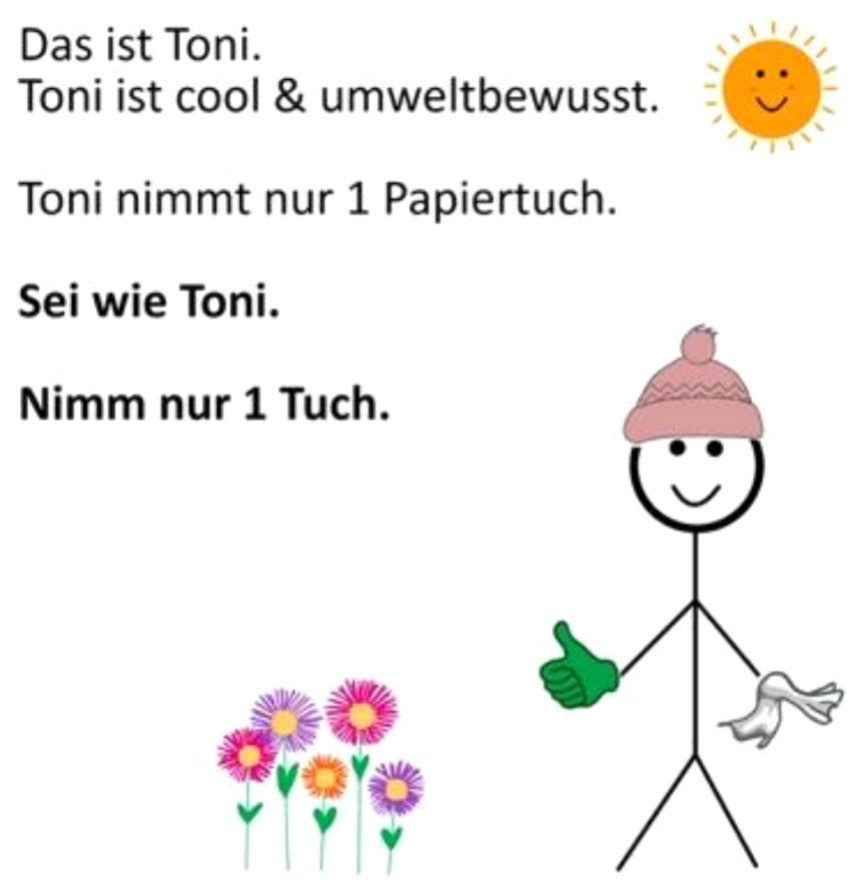
Social norm nudge used in experiment 1.

The intervention was a self-designed nudge. Its design was based on the social media format “Be like Bill” (Croitoru and Nath, 2016), but given the gender-neutral name “Toni.” It was printed in color on paper (13.5 cm x 13.5 cm; 100 g/m2) and laminated in. At the left hand-side of the stick figure was the following text written: “This is Toni. Toni is cool and environmentally conscious. Toni only takes 1 paper towel. Be like Toni. Take 1 tissue only.” (close English translation). The message combines an injunctive norm (“Toni is cool and environmentally conscious.”) and a descriptive norm message (“Toni only takes 1 paper towel.”). The stick figure holds a paper towel in its left hand. The right hand was showing a thumb sticking up. A small sun and flowers were meant to reinforce an environmental association (see Figure 1).

#### 2.1.3 Procedure

The field experiment took place in May 2022 in the women’s restroom located in a learning center at a German University. The restroom consisted of a sink with a large mirror above it, two paper towel dispensers on the left and right, and a trash can positioned to the right of the sink. Additionally, there were three toilet cubicles situated in a separate room, accessible through a door.

In the nudge condition, the nudge-stickers were placed in the center of both paper towel dispensers. In the control condition, the nudge-stickers were taken off. We rotated the condition approximately every 15 people to ensure randomized assignment to conditions and avoid day and time effects. The exact number of people before changing the condition was adjusted if people were still in the restroom at the time of a scheduled condition change. To count the used paper towels for each participant, a female experimenter remained inconspicuously in the restroom. In order not to attract attention, she carried out inconspicuous activities such as braiding hair, cleaning glasses, filling bottles or checking cell phones - behaviors that are frequently observed in women’s toilets and are therefore not considered unusual for the test subjects. An observation period lasted 45 min each time and was followed by a 15-min break before experimenters changed.

### 2.2 Results

Data processing and analysis was conducted in R (version 4.3.2; R Core Team, 2021). As inference criteria, we employed an alpha level of 0.05.

The number of paper towels used ranged from 0 to 9 towels. To test our hypothesis, we ran a one-tailed Welch t-test between two independent means. Participants in the nudge condition used less paper towels (*M* = 2.45, *SD*  = 1.33) than the control condition (*M* = 3.11, *SD* = 1.42), *t*(176.53) = 3.22, *p* < 0.001, *d* = 0.48, confirming our hypothesis.

### 2.3 Discussion

Experiment 1 found participants to use less paper towels when shown our humorous social norm nudge combining descriptive and injunctive social norms compared to participants who were not confronted with any nudge intervention.

In this first experiment, our sample consisted of young, female-presenting academic students. Young adults are often more sustainability-oriented than groups of older people (Yamane and Kaneko, 2021). Therefore, they might be more responsive to our social norm nudge. Moreover, due to their activities in social media, young adults might also be more familiar with the “Be Like Bill”- format than older adults. It is possible that the effect of the nudge also depends on its popularity and therefore, is less effective in older adults. Therefore, in Experiment 2, we tested our nudge with a more heterogeneous sample, not only related to age, but also to other social demographic characteristics, as for instance their educational background. Investigating the nudge also with an educational heterogeneous sample is crucial for further practical recommendations of our used nudge, since our nudge intervention communicates social norms in written format.

## 3 Experiment 2

To explore the nudge’s effectiveness, Experiment 2 extended the same nudge intervention to a more diverse setting, specifically a female restroom at a Christmas market. As the experiment took place outside the university, at an event of widespread interest to the town’s population, we anticipated a more varied sample compared to Experiment 1 regarding age and educational backgrounds. Since the applicability of nudge intervention depends on situational and contextual constraints (Czajkowski et al., 2019; Grilli and Curtis, 2021; Mertens et al., 2022; Dannenberg and Weingärtner, 2023), the examination of the nudge’s effectiveness with a different target group is vital for practical recommendations. We kept the area of application (paper towel use in public women’s toilets) and the nudge itself constant in order to be able to make appropriate statements about the scalability of the nudge intervention regarding other demographic characteristics of the target group. As for experiment 1, we expected participants in the nudge condition to use less paper towels than participants in a control condition (directional).

### 3.1 Method

The study was preregistered before data collection at: https://osf.io/xmnkh.

#### 3.1.1 Design and sample

Our field experiment followed a one-factorial between-subjects design with the independent variable nudge (control condition vs. nudge condition) and the dependent variable amount of used paper towels.

Participants were *N* = 183 female-presenting adults (estimated age range: 19 to 75 years, *M* = 42.51; *SD* = 14.56), with 88 participants in the nudge condition and 95 participants in the control condition. Our data collection included all individuals who washed their hands in the restroom, while excluding those who made multiple restroom visits during the observation period (based on experimenters’ recognition), participants who witnessed the nudge installation, individuals who used paper towels for purposes other than hand drying, and cases where the count of taken paper towels was ambiguous. Participants did not know that they were participating in an experiment.

An *a priori* sample size estimation with G*Power (version 3.1.9.6, Faul et al., 2007) suggested an optimal sample size of 176 participants for a one-tailed independent t-test. We assumed a Type-1 error probability of 0.05, a power of 0.95 and a medium-sized effect of *d* = 0.50.

#### 3.1.2 Procedure

The field experiment was conducted in December 2022 in a public restroom on the Christmas market in a mid-sized German city. Two restroom containers were located next to each other, each container contained three toilets and one sink. On the left side of the sink was the paper towel dispenser, directly over the sink was a small mirror. A small trash bin was located under the paper towel dispenser. The door of the restroom container was open, so that the experimenter could unobtrusively observe the sink from outside of the container. The experimenters disguised as waiting relatives - a not uncommon behavior for restroom visits at such events. By doing so, they did not raise attention by staying in the restroom container without a purpose.

In the nudged condition, the same nudge from Experiment 1 was placed on top of the paper towel dispenser, so that the subject had to look at the nudge while pulling paper towels from the dispenser.

### 3.2 Results

The statistical analysis was executed in R (version 4.3.2; R Core Team, 2021). As inference criteria, we employed an alpha level of 0.05.

The range of used paper towels was from 0 to 6. To test our hypothesis that less paper towels were used in the nudge condition compared to the experimental condition, we ran a one-tailed Welch t-test between two independent means. Confirming our hypothesis, participants in the nudged condition used less paper towels (*M* = 2.19; *SD* = 0.92) than people from the control condition (*M* = 2.54 *SD* = 1.19), *t*(175.42) = 2.19, *p* = 0.015, *d* = 0.32.

### 3.3 Discussion

Experiment 2 successfully replicated the results of Experiment 1, demonstrating that participants who were exposed to a social norm nudge used fewer paper towels compared to those who did not receive any nudge intervention. This indicates that our nudging approach was effective not only with a young academic sample but also with a more diverse sample regarding age and educational background. It should, however, be noted that the effect size in Experiment 2 was smaller than in Experiment 1.

## 4 Experiment 3

Experiment 3 was intended to investigate potential differences in the wording of the nudge with regard to prescriptive or proscriptive norms (see Section 1.1). Prescriptive norms describe a behavior that is normal or appropriate (i.e., “do-norm”) and proscriptive norms refer to behavior that is not normal or inappropriate (i.e., “do not-norm”). So far, there are inconclusive findings concerning their effectiveness in norm interventions, while there is a slight majority of studies supporting the superiority of proscriptive norms (Bergquist and Nilsson, 2019, study 3; Cialdini et al., 2006; Mollen et al., 2021, study 2; Pavey et al., 2018, 2021, study 4). Therefore, testing whether proscriptive norms are more effective than prescriptive norms seems relevant for the present norm nudge as well as for other social norm interventions. Based on the results of Experiments 1 and 2, we generally expected an intervention effect. Thus, compared to participants who were not exposed to any nudge intervention, we expected a lower paper towel use for participants who were exposed to the prescriptive nudge (H1), or respectively, to the proscriptive nudge (H2). Based on the cited literature, we hypothesized that participants who were exposed to a proscriptive nudge consume less paper towels than participants who were exposed to a prescriptive nudge.

### 4.1 Method

The study was preregistered before data collection at: https://osf.io/hzcxw/.

#### 4.1.1 Design and sample

Our field experiment followed a one-factorial between-subjects design with the independent variable nudge (control condition vs. prescriptive social norm nudge vs. proscriptive social norm nudge) and the dependent variable amount of used paper towels.

Participants were *N* = 250 female-presenting adults (estimated age range: 18 to 66 years (*M* = 22.18; *SD* = 4.02)), with 82 participants in the prescriptive norm condition, 82 in the proscriptive norm condition and 86 participants in the control condition. As before, we included everyone in our data collection who washed and dried their hands in the restroom, while excluding those who made multiple bathroom visits during the observation period (based on experimenters’ recognition), participants who witnessed the nudge installation, and individuals who used paper towels for purposes other than hand drying. Participants did not know that they were participating in an experiment.

An *a priori* sample size estimation with G*Power (Version 3.1, Faul et al., 2007) suggested an optimal sample size of 258 participants for three one-tailed independent *t*-tests. Due to Bonferroni correction, we assumed a Type-1 error probability of 0.017, a power of 0.90 and a medium-sized effect of *d* = 0.50.

#### 4.1.2 Material

In our prescriptive and proscriptive nudge condition we used a modified “Be like Toni” meme as it was used in Experiment 1.

Our prescriptive meme showed a smiling stick figure holding a single paper towel in its left hand. Above the head of the stick figure we placed a green thumbs up. On the left of the stick figure a smiling sun and blooming flowers were located to underpin the aspect of environmentally friendly behavior. Additionally, the following text was written:” This is Toni. Toni is cool and environmentally conscious. Toni only takes 1 paper towel. Be like Toni. Take 1 tissue only.” (close English translation). Our proscriptive meme showed a disinterested looking stick figure holding several paper towels in both hands. Above the head of the stick figure we placed a red thumbs down. On the left of the stick figure an unpleasant looking sun and withered flowers were located to underpin the aspect of environmentally harmful behavior. Additionally, the following text was written:” This is Toni. Toni is uncool and harmful to the environment. Toni takes more than one paper towel. Do not be like Toni. Do not take more than one paper towel.” (close English translation).

#### 4.1.3 Procedure

The field experiment was conducted in December 2022, once again in the same female restroom used in Experiment 1, located at a university’s learning center.

In the experimental conditions, the prescriptive or proscriptive nudge-stickers were placed in the center of the paper towel dispenser that was located in the restroom right from the mirror and the sink. In the control condition, the nudge stickers were taken-off. We rotated the condition after every observation period, to ensure randomized assignment to conditions and avoid day and time effects. An observation period lasted 45 min each time and was followed by a 15-min break before the experimenter changed. One of the three female experimenters was present in the restroom to record the quantity of paper towels used by each participant. To avoid drawing attention, the experimenters engaged in activities such as refilling a water bottle, applying makeup, cleaning glasses, or using their phones. In order not to attract attention, the experimenter carried out inconspicuous activities such as refilling bottles or checking cell phones, as in experiment 1 - behaviors that are frequently observed in female restrooms and are therefore perceived as normal by the test subjects. However, they refrained from drying their own hands to prevent any potential influence on the participants’ behavior.

### 4.2 Results

Data processing and analysis was conducted in R (version 4.3.2; R Core Team, 2021). To test our three hypotheses, we run three one-tailed Welch t-tests between two independent conditions. As inference criteria, we employed an alpha level of 0.017, as we applied a Bonferroni correction to adjust our overall alpha level of 0.05.

The number of paper towels used ranged from 1 to 8 towels. Hypothesis 1 was confirmed, as participants in the prescriptive nudge condition (*M* = 2.89, *SD* = 1.31) used fewer paper towels compared to those in the control condition (*M* = 3.50, *SD* = 1.31), *t*(165.54) = −3.01, *p* = 0.002, *d* = 0.46. Also, Hypothesis 2 was supported, as participants in the proscriptive nudge condition (*M* = 2.95, *SD* = 1.44) used fewer paper towels compared to those in the control condition, *t*(162.68) = −2.58, *p* = 0.005, *d* = 0.40.

No difference in paper towels usage was found between participants in the proscriptive nudge condition and the prescriptive nudge condition, *t*(160.69) = −0.28, *p* = 0.777. Thus, we could not confirm Hypothesis 3 that less paper towels were used in the proscriptive nudge condition compared to the prescriptive nudge condition.

### 4.3 Discussion

Experiment 3 replicated the results of Experiments 1 and 2, reaffirming the effectiveness of our social norm nudge intervention in female restrooms to reduce paper towel usage. The study demonstrated that the social norm nudge intervention was effective with both prescriptive and proscriptive norm messages. Yet, no significant difference was observed between prescriptive and prescriptive norms. Hence, the assumed stronger effects for proscriptive norms could not be supported and the following experiments (4 and 5) only applied prescriptive norms. The effect size was similar to Experiment 1, thus stronger than in Experiment 2.

## 5 Experiment 4

To explore the effectiveness of the nudge in different contexts and regarding a different pro-environmental behavior, Experiment 4 examined the impact of our nudge on the usage of plastic lids for disposable cups.

In Germany, approximately 2.8 billion hot beverages consumed in disposable to-go cups result in about 28,000 tons of waste annually. The environmental impact of these to-go cups largely hinges on customers’ decision to use or not to use a plastic lid, with opting for no lid being the more environmentally friendly choice (Kauertz et al., 2019). Several studies speak for social norm nudging as an effective approach to reduce single-use plastic lids for hot beverages to-go. For example, Loschelder et al. (2019) demonstrated the efficacy of norm-based nudging in helping café customers avoid disposable to-go cups. Dorigoni and Bonini (2023) successfully reduced the demand for bottled water in favor of tap water for beverages by implementing a written descriptive norm intervention. Therefore, we hypothesized that customers of the experimental group who were exposed to the nudge are less likely to use a plastic lid for their to-go coffee than customers of the control group who were not exposed to the nudge.

### 5.1 Method

The study was preregistered at: https://osf.io/3knyv.

#### 5.1.1 Design and sample

Our field experiment followed a one-factorial between-subjects design with the independent variable nudge (control condition vs. nudge condition) and the dependent variable lid use (0 = no use, 1 = use).

For data collection, we included everyone who bought a beverage in a to-go cup. A total of 208 participants were included in our data set. Participants did not know that they were participating in an experiment. Two customers who were estimated to be 17 years old were excluded from data analysis. Our final sample were *N* = 206 adults (99 male-presenting, 106 female-presenting, 1 diverse-presenting; estimated age range: 18 to 67 years, *M* = 39.62, *SD* = 13.67), with 102 participants in the experimental condition (48 male-presenting, 54 female-presenting) and 104 participants in the control condition (51 male-presenting, 52 female-presenting, 1 diverse-presenting).

An *a priori* sample size estimation with G*Power (version 3.1.9.6, Faul et al., 2007) suggested an optimal sample size of 208 participants for a one-tailed binomial logistic regression (*z*-test). We assumed a Type-1 error probability of 0.05, a power of 0.95 and an effect size of *OR* = 0.21 (based on estimated probabilities for *p*(H1) = 0.8 and *p*(H0) = 0.95).

#### 5.1.2 Material

Our nudge was similar to the previous experiments, this time depicting a stick figure holding a cup of coffee without plastic lid in its right hand. The text said: ‘This is Toni. Toni is environmentally friendly. Toni goes without lid. Be like Toni. Go without lid.’ (close English translation). The nudge was printed in color on a 20x20cm surface and protected with lamination.

#### 5.1.3 Procedure

The research took place in December 2022 at a self-service café of a long-distance train station in Germany. Two self-service coffee machines were available, with disposable cups being the default choice. Milk, sugar, and plastic lids were accessible at a separate counter either before or after payment. During the intervention, the nudge sign was placed next to the stack of plastic lids at the self-service counter. To ensure random assignment of participants to the two conditions, the researchers removed or installed the nudge sign after approximately every 10 participants, taking precautions to prevent subsequent participants from noticing the change. If any participants did happen to notice the alteration, they were not included in our data collection. In cases where participants purchased multiple cups of coffee, they were classified as lid-users if they used a lid for at least one of the cups. The experimenters sat at a table with a view of the coffee machine and the cup-lid counter.to observe costumers’ choices, attempting to blend in as regular customers, drinking coffee, chatting and playing on their cell phones.

### 5.2 Results

Data processing and analysis was conducted in R (version 4.3.2; R Core Team, 2021). As inference criteria, we employed an alpha level of 0.05.

A lid was taken by 47.12% of the participants in the control condition and by 46.08% in the experimental condition.

To analyze the effect of the nudge intervention on the lid choice, a one-tailed binomial logistic regression was conducted with the predictor condition (0 = control, 1 = experimental) and the criterion lid choice (0 = no lid, 1 = lid). The regression model indicated no statistical difference between both nudge conditions, *B* = −0.04 (SE = 0.28), *z* = −0.15, *p* = 0.881. The model fit was not significant, *χ*^2^ (1) = 0.02, *p* = 0.881, Nagelkerke’s *R^2^* = 0.00.

### 5.3 Discussion

Experiment 4 examined the effectiveness of our social norm nudge aimed at reducing the usage of plastic lids for to-go cups. We were unable to replicate findings of Experiments 1–3, thus, customers exposed to the nudge were not less likely to use a plastic lid compared to those not exposed to the nudge. There are several reasons why the nudge did not work in this context.

In Experiments 1–3, the nudge was introduced in a relatively calm environment, allowing participants time to adapt their behavior to the nudging situation. However, in Experiment 4 conducted at a train station, individuals were likely to be more hurried and concerned about avoiding spills while holding their beverages. Hence, the nudge intervention may have been unsuccessful due to the essential need for lids in a travel setting, highlighting a potential contextual dependency of our nudge intervention. Further, due to the hurry, inattentional blindness (Mack, 2003; Jensen et al., 2011) might have occurred, where something is not noticed unless attention is consciously directed toward it, despite being right in front of one’s eyes. In our case, participants’ attention was maybe focused on catching their train or quickly leaving, diverting their attention from the nudge stand. Moreover, the stress experienced by individuals at a train station may also influence decision-making regarding taking a lid or not. Under stress, humans tend to rely on automatic cognitive processes (Alexander et al., 2007; Starcke and Brand, 2012). In our experimental context, this implies that even if participants noticed the nudge, they may not have had the mental capacity at that moment to fully contemplate its message and change their habits. Consequently, it would be interesting to conduct a replication of this study in a café setting where customers have more time available. This would help investigate whether the time factor played a role in hindering customers from adapting their behavior as observed in the previous study.

## 6 Experiment 5

In Experiment 5, the focus was extended to the context of health messages during the COVID-19 pandemic. Having identified the nudge as an effective behavioral nudge in prior studies, it was now evaluated in its ability to increase voluntary mask wearing.

During the Covid-19 pandemic mask wearing had been identified as one of the most effective behaviors reducing the transmission risk of Covid-19 (e.g., Howard et al., 2021; Zhang and Jin, 2023). A long phase of mandatory mask wearing in Germany ended on 19th March 2022 (COVID-19 Snapshot Monitoring, 2023). As a result, mask wearing rates in Germany strongly dropped (COVID-19 Snapshot Monitoring, 2023) despite health officials still encouraged to wear masks on a voluntary basis (Presseagentur, 2022).

This study seeked to increase voluntary mask wearing in a bakery in a heterogenous sample comprising men and women of different ages during the Covid-19 pandemic. Social norm messaging had been successfully employed in encouraging pandemic health behavior such as hand sanitizer use (Mobekk and Stokke, 2020) and increasing vaccination rates (Zhang and Jin, 2023). For the specific behavior of mask wearing, a longitudinal study found that social norms were important determinants of mask wearing behavior (with descriptive norms being more influential than injunctive norms) (Heiman et al., 2023).

It was therefore expected that presenting the social norm nudge to customers in a bakery increases their voluntary health behavior of wearing a face mask. If social norm nudges positively influence health behavior, customers in the experimental group who were exposed to the nudge are more likely to wear a face mask when entering the bakery than customers in the control group who were not exposed to the nudge.

### 6.1 Method

The study was preregistered before data collection at: https://osf.io/xcnd4.

#### 6.1.1 Design

Our field experiment had a one-factorial between-subjects design with the independent variable nudge (control condition vs. nudge condition) and the dependent variable amount of mask wearing (yes vs. no).

#### 6.1.2 Sample

Participants were *N* = 345 adults (181 male-presenting, 164 female-presenting; estimated age range: 19 to 85 years, *M* = 44.81; *SD* = 14.95), with 161 participants in the experimental condition (105 male-presenting, 79 female-presenting) and 184 participants in the control condition (76 male-presenting, 85 female-presenting).

An *a priori* sample size estimation with G*Power (version 3.1.9.6, Faul et al., 2007) suggested an optimal sample size of 226 participants for a one-tailed binomial logistic regression (*z*-test). We assumed a Type-1 error probability of 0.05, a power of 0.95 and an effect size of *OR* = 2.67 (based on estimated probabilities for *p*(H1) = 0.40 and *p*(H0) = 0.20).

We included all participants that have entered the local bakery. All participants who wore a mask more than two meters away from the nudge before entering the bakery, were excluded from both conditions to ensure that they have perceived the nudge. Subjects under the estimated age of 18 were excluded as well. Participants did not know that they were participating in an experiment.

#### 6.1.3 Material

Our nudge was similar to the previous experiments, this time depicting a stick figure wearing a mask. Above the stick figure, we wrote: ‘This is Toni. Toni is cool and conscientious toward other people. Toni is wearing a mask. Be like Toni. Wear a mask.’ (close English translation). Again, our self-created stick figure puts a thumb up. The nudge was printed on a DIN-A4 note.

#### 6.1.4 Procedure

The field experiment was conducted in December 2022 at a local bakery. The bakery shop was equipped with a counter on the left side at the entrance where the sales assistants could serve their customers. On the right side of the entrance there were seats and tables. The observers sat down on the corner of the bakery shop. To make sure that they do not attract attention, activities such as chatting or drinking coffee were performed like normal customers. During the observations, they were not wearing a mask except when standing up to attach or remove the social norm nudge. The backery staff always wore a mask. In the experimental nudge condition, the social norm nudge was attached with scotch tape at the windshield of the bakery shop entry. In the control condition, the social norm nudge was taken off. After every attachment, the observers sat down again and counted which participants were putting their masks on two meters away before entering the bakery. After every 20 participants the conditions were rotated.

### 6.2 Results

Data processing and analysis was conducted in R (version 4.3.2; R Core Team, 2021). As inference criteria, we employed an alpha level of 0.05.

A mask was put on by 16.85% of the participants in the control condition and by 26.19% in the experimental condition.

To analyze the effect of the nudge intervention on the mask wearing, a one-tailed binomial logistic regression was conducted with the predictor condition (0 = control, 1 = experimental) and the criterion mask wearing (0 = no mask, 1 = mask). The regression model indicated statistical difference between both nudge conditions, *B* = 0.71 (*SE* = 0.26), *z* = 2.71, *p* = 0.007, *OR* = 2.03, *d* = 0.39. The model fit was significant, *χ^2^* (1) = 7.49, *p* = 0.006, McFadden’s pseudo-*R^2^* = 0.02. Thus, the proportion of mask-wearing participants in the nudge condition (29.19%) was significantly higher than in the control condition (16.85%), confirming our hypothesis.

### 6.3 Discussion

Experiment 5 examined the effectiveness of the Be-like-Toni-nudge in the context of health messages. Our hypothesis that customers exposed to the nudge were more likely to wear a face mask when entering a bakery was supported. Experiment 5 thereby showed that the nudge can be scaled up to different types of behavior and is applicable in heterogeneous contexts. Experiment 5 furthermore proved the nudge to be effective in target groups with mixed gender. The effect size was similar to Experiment 2, thus smaller than for Experiment 1 and 3.

It is worth noting that the nudge used in our study may have interacted with latent variables such as self-protection, health concerns, or social desirability, thereby influencing participants’ behavior. While we focused primarily on the observable outcome of mask wearing, future studies should consider the potential interactions between the effectiveness of this nudge intervention and the underlying individual motivation of mask wearing.

## 7 General discussion

With five field experiments, we investigated the applicability of an easy-to-implement social norm nudge, based on the social-media meme “Be like Bill” (Croitoru and Nath, 2016), combining descriptive with injunctive social norm messages. While we could replicate a nudging effect in all three experiments aiming at paper towel reduction (Experiments 1–3) as well as mask wearing (Experiment 5), we could not find a nudging effect concerning plastic lid reduction in a coffee shop at a train station (Experiment 4). The nudge was found to be slightly more effective in homogenous samples (Experiment 1 and Experiment 3) as compared to more heterogeneous samples in terms of age, gender, and educational background (Experiment 2 and Experiment 5).

Acknowledging the limited generalizability of individual nudging interventions (e.g., Czajkowski et al., 2019; Grilli and Curtis, 2021; Mertens et al., 2022; Dannenberg and Weingärtner, 2023), our study aimed at identifying patterns that enhance the generalizability of our nudge intervention across diverse situations. Our nudge intervention is a very cost-effective intervention which, due to its simplicity, can easily be integrated into existing systems and therefore, implemented by practitioners. Our incremental changes from experiment to experiment allow us to systematically test assumptions about the scalability of our nudge intervention. The following recommendations can guide practitioners in tailoring this simple intervention to specific contexts and optimizing its effectiveness.

### 7.1 Applicability and boundaries of the presented social norm nudge

#### 7.1.1 Target behaviors and contexts

We observed effects for both paper towels in public toilets and mask wearing during the Covid-19 pandemic. These findings suggest that our nudging intervention is applicable for different target behaviors and thus can serve as a tool to promote pro-environmental and pro-social behaviors. Further replications in different application areas are needed to substantiate this assumption.

The nudge was found ineffective in reducing the consumption rate of plastic lids of disposable coffee cups. This result can be attributed to the contextual conditions rather than to the applicability of the plastic lids *per se*. It can be assumed that the nudge might be effective when the individual is in a decision situation without time pressure and stress. In Experiment 4, the presence of inattentional blindness (Mack, 2003; Jensen et al., 2011) and heightened stress levels during the decision-making process (Alexander et al., 2007; Starcke and Brand, 2012), exacerbated by the urgency to catch a train, may have caused costumers to overlook or ignore our nudge intervention. Furthermore, the fear of possible negative consequences, such as spilled coffee, during the rush to reach the platform might distract attention from the intended effect of the nudge. Therefore, it is plausible that the intervention on plastic lids did not elicit the desired behavioral change, because of the overwhelming influence of situational factors. Consequently, it is imperative to further explore the intervention’s impact in diverse settings, given that distinct situational contexts can influence the efficacy of such nudging interventions (e.g., Costa-Font et al., 2014; Lehner et al., 2016; Czajkowski et al., 2019). Modest adjustments to study designs, as undertaken in this instance, are therefore of great importance in order to determine the scalability of individual interventions.

#### 7.1.2 Sample

Our study examined people of different ages, genders, educational levels and socio-economic backgrounds, whereby the latter two aspects can be assumed based on the study location (Christmas market and bakery). Since we found intervention effects in more homogenous samples (female university students) as much as for more heterogeneous samples (customers of a city’s Christmas market and of a bakery), we assume that our intervention can be applied to a large proportion of the population. Regarding gender effects, it is important to acknowledge that Experiments 1–3 were conducted exclusively in female restrooms (due to constraints in time and personnel resources). The findings from Experiment 5 indicate that the behavioral nudge might be effective in men’s toilets as well. However, further investigation focussing especially on gender differences is warranted in future studies to validate this observation. Thus, previous research has shown that some nudge interventions appeal more to women, others more to men (e.g., Czap et al., 2018). It is therefore necessary to check whether the nudge intervention also works in male restrooms, or whether men show maybe less or no behavioral change due to reactance, for example. It is noteworthy that the effect sizes observed in the more heterogeneous sample compositions (Experiments 2 and 5) were smaller compared to those in Experiments 1 and 3, which employed more homogeneous samples. This observed variation prompts a more in-depth exploration of the effectiveness of our nudge intervention across diverse settings. On the one hand, the diminished effect sizes could be attributed to several factors. First, varied interpretations and acceptance of social norms depending on sociodemographic variables may have contributed to differences in the effectiveness of nudges among participants (Dannenberg and Weingärtner, 2023). Second, the level of familiarity with the presented meme could influence the effectiveness of the nudge. Third, participants with varying levels of reading proficiency may respond differently to the nudge, affecting its overall effectiveness.

On the other hand, the effectiveness of the nudge may also be influenced by the specific context in which participants were nudged. For example in Experiment 2, the restroom environment at the Christmas Market, characterized by close quarters and colder temperatures, may make the hand-drying experience less comfortable, potentially reducing individuals’ inclination to dry their hands with greater attention. In the case of Experiment 5, the limited time to stop and get a mask from the bag, the general habit of wearing masks, and the availability of free hands to put on the mask could play a role in the effectiveness of nudges during a bakery visit.

Considering this, further investigation into the nuanced interplay between individual characteristics and contextual factors is warranted for a more comprehensive understanding of the scalability of our nudge. Regardless of this, the aspects already found highlight the potential impact of our nudge intervention among diverse sample compositions and varying situational contexts.

#### 7.1.3 Nudge message

To counteract possible reactance induced by the nudge intervention (cf. Lee et al., 2020), we decided to use the humorous social media meme “Be like Bill” (Croitoru and Nath, 2016) as the basis of our intervention. Because of a possible character identification (cf. Festinger, 1954; Hoffner, 2020), we decided to give the stick figure the gender-neutral name Toni instead of keeping the previous name Bill. The necessity of this change remains uncertain; however, the nudge proved effective, even if it deviated slightly from the original. It may be worthwhile to further investigate in future studies whether the choice of name influences the results (at all) and what role the meme’s level of recognition plays in the expression of these effects.

As the question concerning the superiority of positively versus negatively worded norm nudges remains somewhat unclear, we compared the effectiveness of prescriptive and proscriptive norm nudges. The difference between the norm messages showed insignificant, similar to some of the existing research (Leoniak and Maj, 2016; Mollen et al., 2021, study 1). One might conclude based on these results that prescriptive and proscriptive norms are similarly effective. Regarding the mixed results that studies showed, it may also well be that there are moderating variables influencing the effectiveness, which yet have to be investigated. For example, majority social norms might be more effectively communicated via proscriptive norms. Research has shown that proscriptive norms arouse more reactance (Bergquist and Nilsson, 2016) and are linguistically less abstract and therefore potentially more effective (Janoff-Bulman et al., 2009). Therefore, one might assume that in communicating social norms that the majority of people share, proscriptive norms are more effective as reactance is less prevalent in general. For the same reasons, it could be better to communicate minority social norms via prescriptive norms. This remains to be tested.

#### 7.1.4 Temporal effectiveness

Our study focused on short-term interaction effects only, thus how the presentation of our social norm nudge affects an immediate behavioral decision. Therefore, our results do not allow any conclusions regarding the long-term effect of this intervention method. Generally, the state of research concerning the long-term effect of nudging is expandable (Venema et al., 2018). Grilli and Curtis (2021) recommend repeating the presentation of social norm messages over time to achieve long-term effects. For this repeated presentation, we believe our nudge intervention to be very suitable, as its design and wording is simple to adapt and expand.

### 7.2 General limitations and implications

Our study examined the effectiveness of a specific social norm intervention, with the aim of translating findings from behavioral science into a practical tool. It is important to consider that our study did not include any intervention control group. Therefore, we cannot say whether our effects are specific to our social norm nudge or whether other interventions would have been equally effective. Future studies should be devoted to comparing this intervention with other control groups (e.g., including dynamic norms, or normative appeals, Sparkman et al., 2020) to test the uniqueness of our social norm intervention.

Due to our observation method, all our data on gender and age are only estimated, and assumptions on education and socio-economic status were only assumed due to the context and not assessed. Given our data situation, we cannot make any clear statements about the individual effects of the respective socio-demographic characteristics of the sample on the effectiveness of the nudge. In general, confounding with the situation and field of application must be taken into account. Nevertheless, our results provide initial indications of the scalability of the intervention and enable concrete next steps in researching the intervention’s success conditions.

Due to the observation method we have chosen, we cannot completely rule out demand effects that have amplified the nudge effect. However, we took great care to ensure that the experimenters observing the behavior were not conspicuous. Replication with other data collection methods (e.g., recording the average number of paper towels used at the end of a day over a longer period of time) would nevertheless be advisable in order to confirm the effect found.

Finally, our study focused primarily on behaviors that have a low impact on the environment or in the area of health promotion, which, while limiting the scope of interventions addressed, is still useful as these behaviors collectively account for a significant proportion of the overall environmental burden and public health related outcomes. It should also be emphasized that social norm interventions alone cannot be expected to bring about a complete change in behavior. Nudges influence decisions based on intuition rather than addressing the underlying motivation that drives a particular decision-making process (Bhargava and Loewenstein, 2015). However, social norm interventions can help to foster other traditional policy interventions and should be viewed in first place as complements to traditional measures (Benartzi et al., 2017).

## 8 Conclusion

In the present work, five field experiments testing the effectiveness and context dependency of a social norm nudge were presented. The applied social norm nudge Be-Like-Toni has shown to be effective in four of the five field experiments regarding paper towel reduction in a university as well as public restrooms and promoting voluntary mask wearing in a bakery. The social norm nudge has thus shown to be an effective, low-cost and easy-to-implement intervention motivating pro-environmental and pro-social behavior. The social nudge has shown to be implementable in different contexts and thus can be implemented into the real world by practitioners.

## Data availability statement

The datasets presented in this study can be found in online repositories. The names of the repository/repositories and accession number(s) can be found at: https://osf.io/xg4h6/.

## Ethics statement

Ethical review and approval was not required for the study on human participants in accordance with the local legislation and institutional requirements. Written informed consent from the patients/ participants or patients/participants legal guardian/next of kin was not required to participate in this study in accordance with the national legislation and the institutional requirements.

## Author contributions

DM: Writing – review & editing, Writing – original draft, Methodology, Investigation, Formal analysis, Data curation, Conceptualization. MB: Writing – review & editing, Writing – original draft, Methodology, Conceptualization. TB: Writing – original draft, Methodology, Investigation. SG: Writing – original draft, Methodology, Investigation. AH: Writing – review & editing, Writing – original draft, Methodology, Investigation, Formal analysis, Data curation, Conceptualization.

## Footnotes

^1^We would like to point out that our age estimates in all five experiments were based solely on the personal perceptions of our experimenters and not on any specific or standardized procedure.
